# Association of Short-Chain Fatty Acids in the Gut Microbiome With Clinical Response to Treatment With Nivolumab or Pembrolizumab in Patients With Solid Cancer Tumors

**DOI:** 10.1001/jamanetworkopen.2020.2895

**Published:** 2020-04-16

**Authors:** Motoo Nomura, Ryosuke Nagatomo, Keitaro Doi, Juko Shimizu, Kiichiro Baba, Tomoki Saito, Shigemi Matsumoto, Koichi Inoue, Manabu Muto

**Affiliations:** 1 Department of Medical Oncology, Kyoto University Graduate School of Medicine, Kyoto, Japan; 2 Laboratory of Clinical and Analytical Chemistry, Ritsumeikan University College of Pharmaceutical Sciences, Shiga, Japan

## Abstract

**Importance:**

Immunotherapy using immune checkpoint inhibitors has been remarkably effective for treating multiple cancer types, and the gut microbiome is a possible factor affecting immune checkpoint inhibitor efficacy. However, the association between the gut microbiome and immune status of the tumor microenvironment remains unclear. Short-chain fatty acids (SCFAs) are major end product metabolites produced by the gut microbiota and have wide-ranging impacts on host physiology.

**Objective:**

To evaluate fecal and plasma SCFAs in patients with solid cancer tumors treated with programmed cell death-1 inhibitors (PD-1i).

**Design, Setting, and Participants:**

This was a prospective cohort biomarker study of patients with cancer who planned therapy with PD-1i at Kyoto University Hospital between July 2016 and February 2019. Data were analyzed from October 2019 to February 2020.

**Exposures:**

Patients who were treated with nivolumab or pembrolizumab were classified into 2 groups based on their treatment response using Response Evaluation Criteria in Solid Tumors version 1.1: responders who achieved an objective response and nonresponders. Dietary information in terms of intake frequency was obtained. Concentrations of SCFAs in fecal and plasma samples collected before PD-1i administration were measured using ultra-high-performance liquid chromatography coupled with tandem mass spectrometry.

**Main Outcomes and Measures:**

The concentration of SCFAs and progression-free survival.

**Results:**

Among 52 patients enrolled, the median (range) patient age was 67 (27-84) years, and 23 (44%) were women. Median (range) duration of follow-up of the survivors after administration of PD-1i was 2.0 (0.4–4.1) years. The overall response rate was 28.8%. High concentrations of some SCFAs were associated with longer progression-free survival. These included fecal acetic acid (hazard ratio [HR], 0.29; 95% CI, 0.15-0.54), propionic acid (HR, 0.08; 95% CI, 0.03-0.20), butyric acid (HR, 0.31; 95% CI, 0.16-0.60), valeric acid (HR, 0.53; 95% CI, 0.29-0.98), and plasma isovaleric acid (HR, 0.38; 95% CI, 0.14-0.99).

**Conclusions and Relevance:**

Results of this study suggest that fecal SCFA concentrations may associated with PD-1i efficacy; thus, SCFAs may be the link between the gut microbiota and PD-1i efficacy. Because fecal examinations are completely noninvasive, they may be applicable for routine monitoring of patients.

## Key Points

**Question:**

Are short-chain fatty acids associated with clinical outcomes in patients with solid cancer tumors treated with programmed cell death 1 inhibitors?

**Findings:**

In this cohort study of 52 patients with solid tumors, high concentrations of fecal acetic acid, propionic acid, butyric acid, and valeric acid were significantly associated with longer progression-free survival.

**Meaning:**

The findings suggest that fecal short-chain fatty acid concentrations may be a potential biomarker to identify patients with solid tumors who could benefit from treatment with programmed cell death 1 inhibitors.

## Teaser

This cohort study examines the association between fecal and plasma short-chain fatty acid concentrations and response to programmed cell death 1 (PD-1) inhibitors in patients with solid tumors.

## Introduction

Immunotherapy using immune checkpoint inhibitors (ICIs), including programmed cell death 1 inhibitors (PD-1i) and cytotoxic T-lymphocyte antigen 4 inhibitors, given as monotherapies, has consistently demonstrated a long-term survival benefit with durable responses and disease stabilization in patients with untreated or previously treated advanced melanoma.^1,2,3^ Immune checkpoint inhibitors have been remarkably effective across multiple cancer types. However, the response rate of PD-1i for solid cancer was relatively low. An optimal biomarker of the response to ICIs is critically needed for clinical decision-making.

Studies of various tumor types^4,5,6^ have suggested that the gut microbiome profile is a possible factor associated with efficacy of ICIs. Several preclinical and clinical studies have supported an association between the gut microbiome and the efficacy of ICIs, but how this association functions in the tumor microenvironment remains unclear. Short-chain fatty acids (SCFAs) are major end product metabolites produced by the gut microbiota and have wide-ranging impacts on host physiology. The SCFAs have been confirmed to modulate immune cell response. The objective of this study was to evaluate fecal SCFAs in patients with solid cancer tumors treated with a PD-1i.

## Methods

This was a prospective study of patients with cancer who were treated with PD-1i at Kyoto University Hospital between July 2016 and February 2019. A total of 52 patients met the following inclusion criteria: (1) histologically confirmed cancer; (2) age 20 years or older; (3) metastatic or advanced disease with no indication for definitive treatment; (4) planned therapy with PD-1i, specifically nivolumab or pembrolizumab; and (5) written informed consent. The study protocol was approved by the ethics committees and the institutional review boards of Kyoto University Hospital and Ritsumeikan University. This study followed the Strengthening the Reporting of Observational Studies in Epidemiology (STROBE) reporting guideline for cohort studies.

Patients received either nivolumab (2 mg/kg every 3 weeks, 3 mg/kg every 2 weeks, or 240 mg every 2 weeks) or pembrolizumab (200 mg every 3 weeks). All patients were asked about their average frequency and amount of intake of various foods and their dietary habits during the 1 year preceding the onset of their current cancer. Dietary information, including beef or pork, chicken, fish, beans, green vegetables, cabbage, potato, radish, pumpkin, mushroom, seaweed, fruit, and yogurt, was obtained in terms of intake frequency. The following information was obtained from medical records and radiological images: dates of treatment initiation and final date of treatment, age, sex, Eastern Cooperative Oncology Group performance status, primary cancer site, metastatic site(s), laboratory test results at the start of treatment, treatment efficacy according to the Response Evaluation Criteria in Solid Tumors version 1.1, and toxicity according to the Common Terminology Criteria for Adverse Events version 4.0. Patients who were treated with nivolumab or pembrolizumab were followed up using computed tomography approximately every 2 to 3 months and were classified into 2 groups based on their treatment response, according to the Response Evaluation Criteria in Solid Tumors version 1.1. Responders were those who achieved an objective response and nonresponders were those who did not.

### Reagents and Materials

The standards of acetic acid (AA), propionic acid (PA), butyric acid (BA), isobutyric acid (IBA), valeric acid (VA), isovaleric acid (IVA), caproic acid (CA), succinic acid (SA), and methanol were obtained from Fujifilm Wako Chemical Co. 2,2′-Dipyridyl disulfide, triphenylphosphine, 1-ethyl-3-dimethylaminopropyl carbodiimide hydrochloride, and 2-picolylamine, hydroangelic acid and 3-indoleacetic acid were obtained from Tokyo Chemical Co. Propionic acid-*d*_6_, BA-*d*_5_, VA-*d*_9_ and SA-*d*_4_ were obtained from Central Chemicals Co. Caproic acid-*d*_11_ was obtained from Sigma-Aldrich Co. Acetic acid-*d*_4_ and 3-indoleacetic acid-*d*_7_ were obtained from Cambridge Isotope Laboratories Inc. Methanol, acetonitrile, and formic acid were obtained from Fujifilm Wako Chemical Co. Purified water was obtained using a PURELAB flex 5 system (ELGA Co). Stock solutions were adjusted using methanol. Concentrated solutions of mixed standard solutions were diluted as required by adding methanol for derivatization.

### Analysis of SCFAs in Fecal and Plasma Samples

We used the Waters Acquity H Class ultra-performance liquid chromatography system (Waters Co). A reversed-phase analysis was performed via an ultra-performance liquid chromatography BEH C_18_ column (1.7 μm, 2.1 × 100 nm) at 40 °C, with injection volume of 5 μL. The mobile phase, which contained solvent A (0.1% formic acid in water) and solvent B (0.1% formic acid in methanol) was delivered at a flow rate of 0.3 mL/min. The gradient elution was as follows: B% = 2, 2, 10, 10, 35, 98, 98, 2, and 2 (0, 2, 2.1, 2.5, 6, 6.1, 8, 8.1, and 10 minutes). A Waters Xevo TQD triple quadrupole mass spectrometer was operated with electrospray ionization source in the positive mode, with the following ionization source conditions: capillary voltage, 2.00 kV; cone voltage, 30 V; collision energy, 15 eV; source temperature, 150 °C; and desolvation temperature, 400 °C. The cone gas flow was 50 L/h and the desolvation flow was 800 L/h, and they were obtained via a nitrogen source (N_2_ Supplier Model 24S; Anest Iwata Co). Fecal and plasma samples were collected before administration of PD-1 inhibitor and immediately stored at −80 °C.

Fecal samples (approximately 20 mg) after thawing were extracted by 1 mL of methanol, vortexed vigorously and centrifuged at 10 000 rpm for 10 minutes at 4 °C. The feces extraction was diluted by 10-fold in methanol and added to internal standard (10 μL) and QuEChERS (Supel QuE PSA [EN] 25 mg). The solution was vortexed vigorously, homogenized at 500 rpm for 10 minutes, and centrifuged at 10 000 rpm for 10 minutes at 4 °C. The supernatant (100 μL) was then reacted with 2-picolylamine in 2,2′-dipyridyl disulfide and triphenylphosphine in acetonitrile at 60 °C for 10 minutes. The reaction mixtures were removed and redissolved in 100 μL of methanol or water (10:90, v/v). The derivatization solutions (5 μL) were then analyzed by means of ultra-performance liquid chromatography–electrospray tandem mass spectrometry.

For analysis of human plasma, an ultra-performance liquid chromatography BEH C_18_ column (1.7 μm, 2.1 × 150 mm) at 40 °C was used and the mobile phase, which contained solvent A (0.1% formic acid in water) and solvent B (0.1% formic acid in methanol) was delivered at a flow rate of 0.2 mL/min. The gradient elution was as follows: B% = 2, 2, 60, 98, 98, 2, and 2 (0, 2, 9, 9.1, 11, 11.1, and 16 minutes). Plasma samples (25 μL) after thawing were added to internal standard (10 μL) and QuEChERS (Supel QuE PSA [EN] 25 mg), mixed with 1 mL of methanol, vortexed vigorously, homogenized at 1000 rpm for 10 minutes, and centrifuged at 10 000 rpm for 10 minutes at 4 °C. The supernatant was removed and redissolved in 100 μL of methanol. The solution was reacted with 2-picolylamine in acetonitrile and 1-ethyl-3-dimethylaminopropyl carbodiimide hydrochloride in methanol or water (9:1, v/v) and vortexed vigorously. The reaction mixtures were removed and redissolved in 1000 μL of methanol or water (100:900, v/v). Finally, the derivatization solutions (5 μL) were analyzed by means of ultra-performance liquid chromatography–electrospray tandem mass spectrometry.

### Statistical Analysis

All patient characteristics were classified as categorical variables with the exception of age, which was analyzed as a continuous variable. The Mann-Whitney test was performed to evaluate the SCFAs affecting the treatment response. The SCFA concentrations were dichotomized according to the cutoff value using a recursive partitioning analysis. A recursive partitioning analysis was performed to select for the variables in the SCFAs. Dietary information was categorized into 2 groups: less than 3 times/week and 3 times/week or more. Logistic regression analysis was used to obtain odds ratios (ORs) and 95% confidence interval to measure associations. Progression-free survival was defined from the time of treatment initiation to disease progression or death due to other causes. Time-to-event distributions were estimated using the Kaplan-Meier method. To evaluate the impact of each factor on progression-free survival, univariate and multivariate Cox proportional hazards analyses were applied; therefore, the measure of association in this study was the hazard ratio (HR) together with the 95% CI. Variables in multivariate analysis were age (<65 years vs ≥65 years), primary cancer (melanoma vs nonmelanoma), number of organ metastases (<3 vs ≥3), and those that had selected in a recursive partitioning analysis. *P* < .05 using 2-tailed testing was considered indicative of statistical significance. Statistical analyses were performed using Stata version 15 (Stata Corp LP) and R version 3.4.1. (R Project for Statistical Computing). Data were analyzed from October 2019 to February 2020.

## Results

### Patient Characteristics

Among 52 patients enrolled, median (range) patient age was 67 (27-84) years, and 23 (44%) were women (Table 1). Fecal samples were collected from all patients, blood samples from 31 patients, and dietary habits from 48 patients (Figure). Of the 52 patients, 24 (46%) had melanoma, of whom 20 had mucosal melanoma. Of the 24 patients with melanoma, 22 had no *BRAF* mutations, 1 patient with cutaneous melanoma had a *BRAF* mutation, and 1 patient with mucosal melanoma was not evaluated for *BRAF* mutations. Forty-six patients received nivolumab and 6 patients received pembrolizumab. The median (range) follow-up period from PD-1i administration to the time of progressive disease or death was 2.0 (0.7-2.5) years. The median (range) follow-up period of the survivors after administration of PD-1i was 2.0 (0.4-4.1) years. The overall response and disease control rates of all patients were 28.8% and 46.1%, respectively.

**Table 1.** Patient Characteristics

Characteristic	No.
Total	52
Age, median (range), y	67 (27-84)
Sex	
Male	29
Female	23
Eastern Cooperative Oncology Group performance status	
0	28
1	23
2	0
3	1
Body mass index, median (range)^a^	21.1 (13.8-26.9)
**Primary cancer**	
Melanoma	24
Mucosal	20
Cutaneous	2
Uveal	2
Head and neck	10
Squamous cell carcinoma	7
Adenocarcinoma	2
Adenocystic carcinoma	1
Gastrointestinal	9
Squamous cell carcinoma	8
Adenocarcinoma	1
Genitourinary	6
Renal cell carcinoma	4
Urothelial carcinoma	2
Lung, adenocarcinoma	2
Sarcoma, leiomyosarcoma	1
No. of organ metastases	
0	10
1	18
2	13
≥3	11
Treatment	
Nivolumab	46
Pembrolizumab	6
Follow-up period, median (range), y	2.0 (0.4-4.1)

^a^ Calculated as weight in kilograms divided by height in meters squared.

**Figure.**
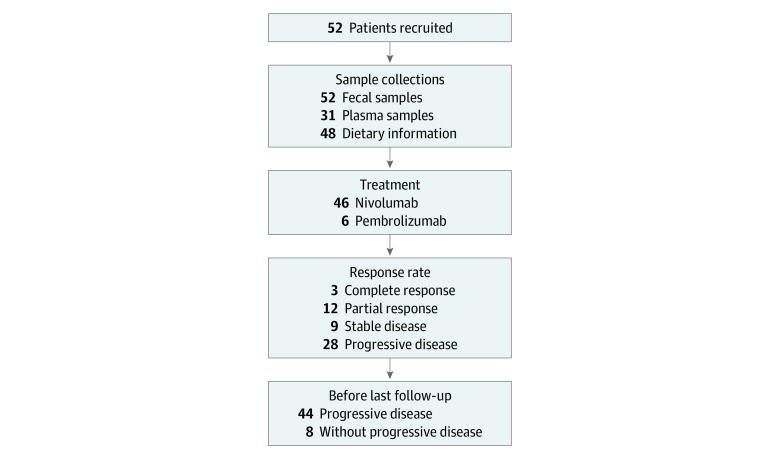
Patient Enrollment, Sample Collections, and Evaluable Population The overall response and disease control rates of all patients were 28.8% and 46.1%, respectively.

### The Associations Between SCFA Concentrations and PD-1i Efficacy

For the analytical validation, our previous study was reported using biological samples.^7^ Patient characteristics and the concentrations of fecal and plasma SCFAs in the responder and nonresponder groups are summarized in Table 2. There were no significant differences between the responder and nonresponder in patient characteristics. The concentrations of fecal and plasma SCFAs were higher in the responder than nonresponder groups. The fecal concentrations of AA, PA, BA, and VA and plasma concentrations of PA and IVA were all significantly higher in the responder than nonresponder groups (Table 2). There was no significant association between each individual fecal and plasma SCFA concentrations according to the Spearman rank correlation coefficient. Univariate analyses of the associations of patient characteristics and each fecal SCFA concentration with progression-free survival revealed 4 variables that were significantly associated with longer progression-free survival: high fecal concentrations of AA (HR, 0.29; 95% CI, 0.15-0.54) PA (HR, 0.08; 95% CI, 0.03-0.20), BA (HR, 0.31; 95% CI, 0.16-0.60), and VA (HR, 0.53; 95% CI, 0.29-0.98) (Table 3). Of the plasma SCFAs, a high concentration of IVA was significantly associated with longer progression-free survival (HR, 0.38; 95% CI, 0.14-0.99) (Table 3). A recursive partitioning analysis that included fecal SCFAs and plasma SCFAs showed that fecal PA was the initial discriminator of progression-free survival. By multivariate analysis, only fecal PA was significantly associated with progression-free survival (HR, 0.07; 95% CI, 0.03-0.19).

**Table 2.** Patient Characteristics and Short-Chain Fatty Acid Concentrations in the Programmed Cell Death 1 Inhibitor Responder and Nonresponder Groups

Characteristic or SCFA	No.		*P* value
	Responder	Nonresponder	
Sex			
Male	8	21	.82^a^
Female	7	16	
Eastern Cooperative Oncology Group performance status			
0	9	19	.57^a^
1-3	6	18	
Primary cancer			
Melanoma	7	17	.96^a^
Nonmelanoma	8	20	
No. of organ metastases			
<3	12	29	.89^a^
Age, median (range), y	73 (39-84)	66 (27-84)	.11^b^
Body mass index, median (range)^c^	22.2 (13.8-26.9)	20.5 (14.3-26.6)	.15^b^
Fecal concentration, median (range), μmol/g			
Acetic acid	346.4 (162.0-652.8)	216.7 (30.3-510.8)	.02^b^
Propionic acid	172.6 (92.2-302.1)	103.6 (1.2-350.8)	.002^b^
Butyric acid	89.0 (20.6-211.3)	40.4 (0.0-218.1)	.004^b^
Isobutyric acid	33.8 (2.9-78.1)	19.0 (0.0-81.5)	.05^b^
Valeric acid	19.7 (0.3-118.8)	9.6 (0.0-45.4)	.04^b^
Isovaleric acid	21.3 (3.7-49.2)	11.2 (0.0-56.1)	.11^b^
Hydroangelic acid	17.3 (1.6-42.0)	10.4 (0.0-34.2)	.36^b^
Caproic acid	0.9 (0.2-13.6)	0.6 (0.0-5.6)	.09^b^
Succinic acid	12.5 (0.0-81.0)	8.4 (0.0-42.3)	.55^b^
Plasma concentration, median (range), μmol/g			
3-Indoleacetic acid	1.1 (0.0-2.0)	1.0 (0.0-17.6)	.83^b^
Propionic acid	5.1 (3.2-12.3)	2.7 (0.0-7.2)	.01^b^
Butyric acid	0.6 (0.0-4.1)	0.0 (0.0-3.6)	.12^b^
Isobutyric acid	1.4 (0.0-4.5)	0.8 (0.0-3.8)	.69^b^
Valeric acid	0.0 (0.0-0.9)	0.0 (0.0-0.6)	.37^b^
Isovaleric acid	0.3 (0.0-0.9)	0.0 (0.0-0.4)	.01^b^
Hydroangelic acid	0.0 (0.0-1.3)	0.0 (0.0-1.3)	.49^b^
Caproic acid	1.6 (0.9-3.2)	1.2 (0.0-3.6)	.42^b^
Succinic acid	0.0 (0.0-6.3)	0.0 (0.0-15.3)	.63^b^

Abbreviations: SCFA, short-chain fatty acid.

^a^ Calculated using a χ^2^ test.

^b^ Calculated using a Mann-Whitney test.

^c^ Calculated as weight in kilograms divided by height in meters squared.

**Table 3.** Factors Associated With Progression-Free Survival After Programmed Cell Death 1 Inhibitor Treatment

Characteristic or SCFA	HR (95% CI)	*P* value
Age, y		
<65	1 [Reference]	.91
≥65	1.03 (0.56-1.89)	
Sex		
Male	1 [Reference]	.39
Female	0.76 (0.41-1.40)	
Body mass index^a^		
<22	1 [Reference]	.22
≥22	0.67 (0.36-1.26)	
Primary cancer		
Melanoma	1 [Reference]	.30
Nonmelanoma	1.37 (0.75-2.51)	
No. of organ metastases		
<3	1 [Reference]	.46
≥3	1.30 (0.64-2.65)	
Fecal concentration, μmol/g		
Acetic acid		
<270	1 [Reference]	<.001
≥270	0.29 (0.15-0.54)	
Propionic acid		
<90	1 [Reference]	<.001
≥90	0.08 (0.03-0.20)	
Butyric acid		
<40	1 [Reference]	<.001
≥40	0.31 (0.16-0.60)	
Isobutyric acid		
<30	1 [Reference]	.50
≥30	0.80 (0.41-1.54)	
Valeric acid		
<15	1 [Reference]	.04
≥15	0.53 (0.29-0.98)	
Isovaleric acid		
<20	1 [Reference]	.40
≥20	0.76 (0.40-1.43)	
Hydroangelic acid		
<17	1 [Reference]	.62
≥17	0.85 (0.46-1.58)	
Caproic acid		
<2	1 [Reference]	.08
≥2	0.46 (0.19-1.11)	
Succinic acid		
<9	1 [Reference]	.94
≥9	0.97 (0.53-1.77)	
Plasma concentration, μmol/g		
3-Indoleacetic acid		
<1.35	1 [Reference]	.56
≥1.35	0.79 (0.36-1.74)	
Propionic acid		
<6	1 [Reference]	.09
≥6	0.59 (0.32-1.08)	
Butyric acid		
<1.25	1 [Reference]	.82
≥1.25	0.93 (0.51-1.70)	
Isobutyric acid		
<2.55	1 [Reference]	.12
≥2.55	0.62 (0.34-1.14)	
Valeric acid		
<0.1	1 [Reference]	.72
≥0.1	1.17 (0.46-2.97)	
Isovaleric acid		
<0.15	1 [Reference]	.04
≥0.15	0.38 (0.14-0.99)	
Hydroangelic acid		
<0.15	1 [Reference]	.36
≥0.15	0.65 (0.26-1.63)	
Caproic acid		
<1.15	1 [Reference]	.57
≥1.15	0.79 (0.36-1.74)	
Succinic acid		
<2	1 [Reference]	.12
≥2	2.07 (0.82-5.18)	

Abbreviations: HR, hazard ratio; SCFA, short-chain fatty.

^a^ Calculated as weight in kilograms divided by height in meters squared.

### The Associations Between SCFA Concentrations and Dietary Habits

The ORs for the associations between fecal SCFA concentrations and dietary intake are shown in Table 4. The fecal BA concentration was significantly associated with a high frequency of mushroom intake (OR, 6.17; 95% CI, 1.20-31.7); the fecal VA concentration was significantly associated with high frequencies of green vegetable intake (OR, 4.50; 95% CI, 1.30-15.5), cabbage intake (OR, 3.76; 95% CI, 1.01-13.9), and mushroom intake (OR, 3.66; 95% CI, 1.04-12.9); and the fecal hydroangelic acid concentration was significantly associated with a high frequency of green vegetable intake (OR, 4.25; 95% CI, 1.19-15.1). There was no significant association of the fecal concentration of AA, PA, IBA, IVA, CA, or SA with the intake of any food evaluated.

**Table 4.** Association of Dietary Information With Fecal SCFA Concentrations and Progression-Free Survival

Food consumed	Odds ratio (95% CI)^a^									Progression-free survival	
	AA	PA	BA	IBA	VA	IVA	HA	CA	SA	Hazard ratio (95% CI)	*P* value
Beef or pork	1.69 (0.37-7.59)	2.08 (0.38-11.2)	0.78 (0.18-3.26)	0.61 (0.11-3.36)	1.53 (0.37-6.21)	0.48 (0.08-2.60)	0.82 (0.18-3.72)	3.64 (0.66-20.0)	1.11 (0.27-4.47)	0.43 (0.17-1.03)	.06
Chicken	0.60 (0.13-2.76)	0.71 (0.14-3.47)	0.89 (0.18-4.32)	1.80 (0.36-8.91)	2.77 (0.57-13.3)	1.40 (0.28-6.81)	2.33 (0.49-10.9)	5.40 (0.92-31.5)	2.03 (0.42-9.70)	1.11 (0.49-2.53)	.79
Fish	0.84 (0.26-2.67)	1.80 (0.52-6.21)	1.20 (0.36-3.92)	1.23 (0.34-4.42)	2.00 (0.62-6.42)	0.82 (0.24-2.79)	0.46 (0.13-1.59)	2.89 (0.50-16.6)	2.33 (0.73-7.42)	0.70 (0.37-1.32)	.27
Bean	1.19 (0.29-4.80)	2.43 (0.45-13.0)	0.94 (0.23-3.85)	1.77 (0.42-7.50)	1.22 (0.31-4.74)	1.35 (0.32-5.56)	1.96 (0.49-7.83)	0.51 (0.05-4.82)	0.54 (0.13-2.16)	0.91 (0.43-1.94)	.82
Green vegetables	0.58 (0.17-1.90)	1.47 (0.41-5.28)	1.97 (0.56-6.97)	3.49 (0.92-13.1)	4.50 (1.30-15.5)	3.45 (0.96-12.3)	4.25 (1.19-15.1)	2.31 (0.45-11.7)	0.96 (0.30-3.07)	0.83 (0.43-1.59)	.58
Cabbage	0.54 (0.15-1.92)	1.19 (0.30-4.67)	0.98 (0.26-3.60)	1.80 (0.46-6.96)	3.76 (1.01-13.9)	2.08 (0.56-7.67)	1.16 (0.31-4.29)	2.04 (0.39-10.6)	0.31 (0.08-1.20)	0.79 (0.40-1.56)	.50
Potato	0.34 (0.08-1.45)	1.07 (0.23-4.90)	1.36 (0.30-6.12)	1.20 (0.25-5.55)	1.53 (0.37-6.21)	1.63 (0.38-6.95)	1.44 (0.34-6.08)	0.59 (0.06-5.58)	1.11 (0.27-4.47)	0.67 (0.29-1.56)	.36
Radish	0.82 (0.23-2.92)	3.71 (0.71-19.3)	2.56 (0.60-10.9)	1.11 (0.27-4.44)	2.44 (0.68-8.71)	0.50 (0.11-2.14)	0.73 (0.18-2.84)	0.35 (0.03-3.29)	1.68 (0.48-5.93)	0.68 (0.33-1.36)	.28
Pumpkin	1.36 (0.22-8.27)	2.49 (0.26-23.5)	NA	3.20 (0.55-18.4)	1.47 (0.26-8.17)	2.50 (0.44-14.1)	2.23 (0.39-12.5)	1.20 (0.11-12.1)	6.66 (0.71-62.1)	0.58 (0.20-1.65)	.31
Mushroom	0.77 (0.22-2.61)	4.78 (0.92-24.7)	6.17 (1.20-31.7)	2.14 (0.57-7.97)	3.66 (1.04-12.9)	2.33 (0.65-8.31)	1.32 (0.37-4.64)	3.22 (0.62-16.6)	0.77 (0.23-2.59)]	0.40 (0.19-0.81)	.01
Seaweed	0.44 (0.10-1.94)	1.75 (0.31-9.64)	2.18 (0.40-11.9)	1.45 (0.30-6.90)	2.00 (0.46-8.65)	1.12 (0.24-5.26)	0.78 (0.21-4.65)	0.68 (0.07-6.54)	2.58 (0.56-11.8)	0.71 (0.31-1.62)	.42
Fruit	0.78 (0.24-2.51)	1.88 (0.52-6.71)	1.71 (0.50-5.81)	2.70 (0.72-10.0)	1.09 (0.34-3.45)	1.18 (0.34-4.05)	1.41 (0.43-4.88)	095 (0.18-4.83)	2.76 (0.85-8.96)	0.65 (0.34-1.25)	.20
Yogurt	1.47 (0.41-5.28)	0.56 (0.15-2.03)	1.14 (0.31-4.13)	0.96 (0.24-3.84)	0.60 (0.16-2.14)	0.43 (0.10-1.86)	0.63 (0.16-2.44)	NA	2.03 (0.58-7.05)	0.64 (0.31-1.33)	.24

Abbreviations: AA, acetic acid; BA, butyric acid; CA, caproic acid; HA, hydroangelic acid; IBA, isobutyric acid; IVA, isovaleric acid; NA, not available; PA, propionic acid; SA, succinic acid; SCFA, short-chain fatty acid; VA, valeric acid.

^a^ Odds ratios are shown for consumption of each food 3 times per week or more compared with fewer than 3 times per week (reference).

A high frequency of mushroom intake was significantly associated with longer progression-free survival (HR, 0.40; 95% CI, 0.19-0.81) (Table 4). There was no significant association between green vegetable or cabbage intake and progression-free survival (green vegetable: HR, 0.83; 95% CI, 0.43-1.59; cabbage: HR, 0.79; 95% CI, 0.40-1.56).

## Discussion

To our knowledge, this is the first study to find an association between SCFAs and the efficacy of PD-1i treatment for solid cancers. Several reports explored gut microbiome profiling in association with the response to anti-PD-1 antibody treatment in patients with various solid tumors. Although the mechanisms of the association between the gut microbiome and the response to ICIs remain unclear, gut microbial metabolites are thought to link the gut microbiome to systemic immunity. Short-chain fatty acids are the end products of gut microbe-mediated metabolism and the most abundant product of anaerobic fermentation of dietary fibers in the intestine.

Several reports have evaluated gut microbiome profiling in association with the response to PD-1i treatment, but the results differ among those reports.^4,5,6^ Even 2 of those studies^5,6^ that were conducted in the same country, both in melanoma patients, identified different bacteria species associated with the response to PD-1i treatment. However, the species associated with the PD-1i response, such as *Faecalibacterium prausnitzii*, *Akkermansia muciniphila*, *Collinsella aerofaciens*, and *Bifidobacterium adolescentis*, are BA- or PA-producing bacteria.^8,9,10^

Our study found that high concentrations of fecal or plasma SCFAs were associated with a response to PD-1i treatment and longer progression-free survival. Short-chain fatty acids exhibit immunomodulatory functions in the host, affecting CD4^+^ T cells and antigen-presenting cells. Butyric acid has been shown to induce FOXP3^+^CD4^+^ Treg differentiation.^11,12,13^ However, a recent report found that BA and other SCFAs increase the expression of IFNγ and granzyme B in CD8^+^ cytotoxic T lymphocytes and interleukin-17–secreting CD8^+^ T cells.^14^ Another recent report showed that SCFA administration led to the recovery of an impaired immune response.^15^ Another function of SCFAs, particularly BA and VA, is inhibition of histone deacetylases^14,16,17^; histone deacetylase inhibition was found to upregulate PD-1 ligands in melanoma cells, enhance the immunotherapy response, inhibit apoptosis of CD4^+^ T cells within tumors, upregulate the antitumor immune response, and suppress tumor growth.^18,19,20^ Histone deacetylase inhibitors have been shown to possess potent immunomodulatory activity. The combination of an ICI and histone deacetylase inhibitors has shown promising results.^21,22^

Fecal SCFAs are rapidly absorbed by the colon in a concentration-dependent manner. Butyric acid, in particular, is a local nutrient for mucosal cells. Propionic acid is absorbed by the portal vein and mainly used by the liver as a major hepatic gluconeogenic substrate.^23^ Thus, the concentrations of SCFAs in the peripheral serum are lower than those in the hepatic vein or portal vein.^24,25^

Dietary intake is closely related to fecal excretion of several substances. The composition of gut microbiome changes by changing the food components and dietary habits.^26^ Several studies reported that the gut microbiome depends on many factors, including dietary components, dietary habits, seasonality, and lifestyle.^26,27,28,29^ Fecal concentrations of SCFAs, produced from dietary fibers by the gut microbiota, are also dependent on dietary components and habits.^29,30^ It was unknown which dietary components were related to fecal SCFAs in patients with solid cancer tumors. Our results showed that high frequencies of intake of several sources of dietary fiber, such as green vegetables, cabbage, and mushrooms, were associated with high concentrations of fecal SCFAs. Furthermore, a high frequency of mushroom intake was associated with longer progression-free survival. However, dietary information in this study was not during treatment using ICI, and was during the 1 year preceding the onset of their current cancer. Further study is needed in order to clarify the association between dietary intake before or after treatment and fecal SCFAs or the efficacy of ICIs.

### Limitations

This study has limitations, including the small sample size, lack of a validation cohort, and the limited number of gut microbial metabolites evaluated. The gut microbiome, including the species and diversity, were not analyzed, and consequently, the associations of the gut microbiome with SCFAs were not studied.

## Conclusions

The results of this cohort study suggest that fecal SCFA concentrations are associated with the efficacy of PD-1i treatment; thus, SCFAs may be the link between the gut microbiota and PD-1i efficacy. Because fecal examinations are completely noninvasive, they may be applicable for routine monitoring of patients. Elucidation of the mechanism of SCFA-associated immune modulation could allow the discovery of novel targets to improve PD-1i efficacy in patients with cancer. Additional investigations as validation studies are needed to confirm our findings.
